# Evaluation of nonlinear frequency compression: Clinical outcomes

**DOI:** 10.1080/14992020902971349

**Published:** 2009-06-05

**Authors:** Danielle Glista, Susan Scollie, Marlene Bagatto, Richard Seewald, Vijay Parsa, Andrew Johnson

**Affiliations:** * National Centre for Audiology, Faculty of Health Sciences, University of Western Ontario, Canada; § Bachelor of Health Sciences Program, Faculty of Health Sciences, University of Western Ontario, Canada

**Keywords:** Hearing loss, Hearing aids, Digital signal processing, Frequency compression, Treatment outcome, Clinical trial, Speech detection threshold, Speech perception, Children

## Abstract

This study evaluated prototype multichannel nonlinear frequency compression (NFC) signal processing on listeners with high-frequency hearing loss. This signal processor applies NFC above a cut-off frequency. The participants were hearing-impaired adults (13) and children (11) with sloping, high-frequency hearing loss. Multiple outcome measures were repeated using a modified withdrawal design. These included speech sound detection, speech recognition, and self-reported preference measures. Group level results provide evidence of significant improvement of consonant and plural recognition when NFC was enabled. Vowel recognition did not change significantly. Analysis of individual results allowed for exploration of individual factors contributing to benefit received from NFC processing. Findings suggest that NFC processing can improve high frequency speech detection and speech recognition ability for adult and child listeners. Variability in individual outcomes related to factors such as degree and configuration of hearing loss, age of participant, and type of outcome measure.

Hearing aid technology provides hearing-impaired (HI) individuals with level- and frequency-dependent amplification. For most hearing aid users, hearing aids provide the most gain at higher frequencies, because hearing loss tends to increase with frequency. Current hearing aids have a limited ability to provide sufficient gain for less intense high-frequency sounds (Stelmachowicz et al, 2004). This limits the audibility of high-frequency sounds, particularly for individuals with sloping and/or severe to profound hearing losses. High-frequency speech energy provides listeners with important linguistic information. For example, speech sounds such as /s/ and /z/ are important grammatical markers that denote plurality and possession in the English language (Stelmachowicz et al, 2004). A reliable cue used to perceive the phoneme /s/ is the frequency of the frication (Newman, 2003). Specifically, the spectral peak frequency for the phoneme /s/ is at approximately 5 kHz for male speech, 6 to 9 kHz for female speech, and 9 kHz for child speech (Boothroyd & Medwetsky, 1992; Stelmachowicz et al, 2001). Therefore, consideration should be given to the hearing aid pass-band when attempting to provide audibility of high-frequency speech cues.

Large variability in aided listening performance is often observed for individuals with severe to profound, high-frequency hearing loss. Individual performance with hearing aids can be influenced by both the audibility of high-frequency signals, as well as the listener's proficiency in extracting useful information from the audible signals. Providing audibility through amplification in the high-frequencies for severe or profound HI listeners still remains a controversial topic. Some studies on the relationship between audibility and speech recognition suggest that providing audibility at frequencies where a hearing impairment is severe provides little or no speech recognition benefit; this is thought to be due to limitations in the perceptual abilities of the listener in extracting information from high-frequency energy (Ching et al, 1998, 2001; Hogan & Turner, 1998). Other studies have demonstrated that providing high-frequency information to listeners with sloping sensorineural hearing loss can significantly improve speech understanding, especially when in noisy listening environments (Plyler & Fleck, 2006; Turner & Henry, 2002). Individual performance in such studies indicates that listeners receive varying degrees of speech recognition benefit from amplified high-frequencies. Therefore, it may be necessary to determine efficacy of high-frequency audibility on an individual basis.

Several studies have found significant adult/child differences in the bandwidth required for accurate fricative recognition for listeners with moderate to moderately-severe hearing loss (Pittman & Stelmachowicz, 2000; Stelmachowicz et al, 2001, 2004). In these studies, children required greater high-frequency bandwidth than adults (i.e. above 5 kHz) to achieve similar speech recognition scores for the phoneme /s/ (Stelmachowicz et al, 2001). This suggests that children require audibility of a broad bandwidth of speech for optimal access to fricatives. High-frequency audibility is related to speech perception and production abilities, as well as overall language learning ability. Children provided with extended bandwidth perform better on short-term word learning tasks than children who are provided with a limited bandwidth, regardless of hearing status (Pittman, 2008). Furthermore, infants with hearing loss show a significant delay in fricative and affricate production (Moeller et al, 2007; Stelmachowicz et al, 2004). Children with hearing loss who communicate with female caregivers may therefore experience inconsistent exposure to the spectral cues for /s/, important when forming language-based rules and when learning to monitor their own speech (Stelmachowicz et al, 2002; Pittman et al, 2003).

Frequency-lowering has been suggested as one possible alternative to overcoming bandwidth limitations (Stelmachowicz et al, 2004). The terminology associated with frequency-lowering in hearing aids varies and is not standardized. For the purposes of this paper, we will categorize frequency-lowering technologies into two groups: frequency transposing devices and frequency compressing devices.

Frequency transposition shifts high-frequency sounds to lower frequency regions by a fixed amount. Early attempts at frequency transposition technology included the use of a modulated carrier frequency (Johansson, 1961, 1966) and slow playback speeds to present high-frequency signals at a lower frequency (Beasley et al, 1976; Bennett & Byers, 1967; Ling & Druz, 1967). These early attempts at frequency transposition were somewhat successful in improving speech recognition, but produced unwanted sound quality degradations. Further research by Turner and Hurtig (1999) examined speech recognition scores using a frequency–lowering processor labelled as frequency compression. However, it maintained proportional relations between the formant peaks for a given speech sound, and will therefore be classified as a transposer in this paper. Results suggested significant speech recognition improvement in the transposed condition for approximately half of the adult subjects, with greater benefit for the listeners with more steeply sloping audiograms (i.e. better hearing below 1–2 kHz). Transposition technology has also been evaluated on listeners with suspected dead regions along the basilar membrane; results suggest fricative identification improvement for the phoneme /s/ for listeners with dead regions who were individually fitted with a laboratory transposer (Robinson et al, 2007).

The AVR TranSonic FT-40 was the first commercially available transposition device. This device used a processing unit to analyse incoming signals and apply frequency-lowering to sounds with predominantly high-frequency energy (i.e. above 2.5 kHz). Early studies indicated mixed outcomes with the body-worn FT-40 on adults and children, concluding that the FT-40 system was suitable for a select group of listeners (MacArdle et al, 2001; Parent et al, 1997). AVR Sonovations later introduced the ImpaCt behind-the-ear (BTE) hearing aid. McDermott and Knight (2001) found limited benefit attributable to the ImpaCt transposition signal processing when evaluated on adult listeners; age, training, and audiometric configuration may have contributed to the results. The ImpaCt has also been evaluated on children in a study by Miller-Hansen et al (2003), suggesting significant word recognition benefit could be achieved for children with severe hearing loss when using transposing hearing aids, in comparison to conventional hearing aids.

The Widex Inteo device utilizes spectral analysis to identify the frequency region with peak intensity above a cut-off frequency (i.e. peak frequency). Field studies indicated that 33% of subjects (N = 16), with sloping high-frequency hearing loss preferred listening to conversational speech with the transposer enabled than without (Kuk et al, 2006). Case studies indicated speech recognition improvement for individuals with high-frequency hearing loss wearing the Inteo, in comparison to participants' previously used hearing aids (Auriemmo et al, 2008).

Frequency compression is an alternative frequency-lowering technology. This technology compresses the output bandwidth of the signal by a specified ratio. If applied across the entire frequency range of the device, frequency compression can alter the positions of vowel formants in the frequency domain. Therefore, recent attempts at frequency compression have used a multichannel approach rather than a single-channel approach (Simpson et al, 2005). This strategy applies nonlinear frequency compression (NFC) only to the high-frequency band, preserving the natural formant ratios in the low band. Simpson et al (2005, 2006) used an adjustable cut-off frequency between the high and low bands, and an adjustable frequency compression ratio in the high band. These two parameters were individually fitted per participant.

Simpson et al (2005) reported significant improvements in speech recognition for eight of the seventeen participants with the experimental NFC device. Information on place and voicing cues were made more available to listeners when NFC was used (Simpson et al, 2005). Further research examined the effects of NFC on adult listeners with steeply sloping audiograms (Simpson et al, 2006). No significant benefit in performance was demonstrated in group mean scores when comparing results with NFC to conventional technology; however, subjective preference for the sound quality was noted for conventional technology.

In summary, current conventional hearing aid technology is limited in bandwidth, thereby limiting consistent audibility of high-frequency sound. This has specific detrimental effects on speech sound recognition, particularly in children, but also in adults. This may explain, at least in part, the delay in high-frequency speech sound production observed in children with hearing impairment (Stelmachowicz et al, 2004). Two classes of frequency-lowering technology (i.e. frequency transposition and frequency compression) have been proposed to lower the frequency content of sound in an attempt to overcome this limitation. Such technologies are in the early stages of development and have not been extensively evaluated, particularly in the paediatric population. The purpose of this study was to evaluate one such technology, multichannel NFC, in both children and adults. This investigation provided wearable hearing aids employing NFC processing to HI listeners, and tested both laboratory outcomes (speech recognition) and real world outcomes (functional performance and preference) with and without the NFC processor activated. Results will be presented for group-level and individual-level outcomes.

## Method

### Participants

A total of 24 hearing-impaired participants were included; 13 adults (ages 50–81 years, M = 69) and 11 children (ages 6–17 years, M = 11). Participants were recruited from The University of Western Ontario (UWO) H.A. Leeper Speech and Hearing Clinic, as well as from local audiology clinics and schools. Pure–tone air and bone conduction thresholds were measured bilaterally at all octave and inter-octave frequencies between 250 and 6000 Hz for each participant using a Grason-Stadler 61 audiometer. Air conduction thresholds were obtained using Etymotic Research ER-3A insert earphones coupled to each participant's personal earmolds. Bone conduction thresholds were obtained using a Radioear B-71 bone oscillator. Audiometric testing was completed in a double-walled sound booth. Participants presented with sloping high-frequency hearing losses that ranged from moderately severe to profound in the better ear, and were sensorineural in nature for all but one participant with a mixed loss.

**Figure 1:**
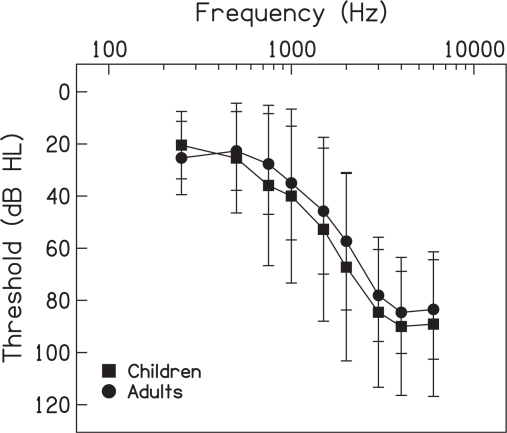
Mean better ear pure-tone thresholds ±1 standard deviation for adults and children.

Figure 1 presents air conduction thresholds for the children and adults. A repeated measures analysis of variance (ANOVA) was completed using audiometric threshold as the dependent variable, repeated across nine frequencies from 250 to 6000 Hz, and age group (adults versus children) as the between-subjects variable. Results did not provide evidence of a significant interaction between audiometric frequency and age group (F(8,15) = .75, p = .65). All hearing losses were symmetrical, with the exception of one subject (2062) who had differences in the range of 10–30 dB between ears in the 2–4 kHz region. Previous hearing aid use included mostly digital hearing aid technology, with three participants having no previous experience with hearing aids and three having previous use of analog hearing aid technology (see Table 3). Participants were evaluated for suspected dead regions using a CD version of the threshold equalizing noise (TEN) test calibrated in dB HL (Moore et al, 2004).

### Trial design

This study used a modified withdrawal design, with both single- and double-blind outcome measures, as follows (Table 1). Participants completed the initial trial, allowing for familiarization to the study worn hearing aid programmed with conventional processing (CP). Following this trial, the outcome test battery was to familiarize participants to the tasks. NFC processing was then enabled and a real world trial allowed participants to familiarize to the NFC processor, and the outcome test battery was administered again. In the final phase of the study, NFC was made optional (via a multimemory fitting) for real world usage, allowing measurement of subjective preference. Allocation of frequency compression to memories was counterbalanced across participants. Prior to re-administering the outcome test battery, NFC was disabled during laboratory measurement, allowing for evaluation of the withdrawal-of-treatment condition; therefore, the objective outcome measures adhered to a true ‘withdrawal’ design while the real world measures of preference did not. Table 2 provides a description of the durations of each phase for each participant. These varied due to scheduling limitations of the participants (e.g. illness, travel).

**Table 1:** Description of the time course of each phase included in the study, corresponding objectives, and phase duration. CP refers to ‘conventional processing’ (i.e. fitting assessment without NFC enabled). Treatment assessment was completed with NFC enabled in the same hearing aid device used for CP evaluation.

Time course	Objective	Duration
Participant intake	Audiometric evaluation. Hearing aid fitting (CP).	Range: 2 weeks to 3 months
Acclimatization phase	Real world trial with CP. Practice tests.	Mean: 4.17 weeks
NFC phase	Real world trial with NFC.	Range: 3 weeks to 1.3 years
	Outcome evaluation with NFC.	Mean: 10.75 weeks
Multimemory phase	Real world trial with user selectable NFC.	Range: 2 weeks to 5 months
	Evaluation of real world preferences.	Mean: 5.58 weeks
Withdrawal testing	Outcome evaluation without NFC.	

**Table 2:** Individual participant hearing aid fitting schedule and corresponding adaption times, expressed in weeks.

Subject	Acclimatization to CP	Treatment (NFC)	Treatment withdrawal (CP)
*Adults*			
1100	3	4	8
1112	3	4	4
1115	4	12	4
1109	4	6	4
1114	2	4	18
1101	4	20	8
1111	5	4	4
1104	6	20	8
1113	3	4	4
1116	4	4	4
1108	4	20	8
1105	5	44	4
1110	4	4	4
*Children*			
2061	12	3	8
2068	4	4	4
2029	3	4	2
2032	3	60	4
2069	4	3	2
2062	4	4	4
2034	4	4	4
2063	4	12	8
2066	4	4	4
2060	3	4	8
2065	4	6	4

A withdrawal study includes three measurement intervals: baseline, treatment, and withdrawal of treatment. In this study, the comparison of interest is benefit with NFC versus benefit without NFC. This was scored using the withdrawal versus treatment intervals (see Results section for details). The withdrawal versus treatment comparison loads any advantage due to practice and acclimatization effects on the withdrawal condition. Therefore, this study employed a more stringent evaluation of NFC benefit, compared to a baseline versus treatment comparison.

Blinding in hearing aid efficacy research is necessary to avoid spurious labelling effects that are akin to placebo effects (Bentler et al, 2003). In the present study, two blinding techniques were used. For computer-administered tests of speech detection and/or recognition, study participants were unaware of the status of the hearing aid signal processing (i.e. single blinding). During experimenter-administered tests of real-world preference, both the participants and experimenters were unaware which condition denoted treatment versus the control (i.e. double blinding). The nature of the NFC processor was not disclosed to the participants at study onset. Participants remained naive to the treatment condition, with the exception of two subjects (1104, and the parent of 2066). These two participants had advanced educational/professional backgrounds in related areas and specifically expressed an understanding of the nature of the treatment based on listening to the hearing aids. No other participants expressed this type of awareness. All participants were debriefed as to the nature of the signal processing upon exiting the study.

### Device fitting without NFC

For the CP hearing aid fittings, the prescriptive targets and clinical protocols from the desired sensation level (DSL) method version 5.0 were used (DSL v5: Bagatto et al, 2005; Scollie et al, 2005). Prototype BTE hearing aids (similar to Phonak Savia 311 or 411, allocated per hearing level, see Table 3) were provided to each participant along with FM compatible audio-shoes. Digital noise reduction features and automatic program selectors were disabled.

**Table 3:** Case history information including previous hearing aid use and conventional make/model of hearing aid fitted for the purpose of the study. Hearing instruments used were prototype versions of the algorithm implemented in current Savia 311/411 hearing aids.

Subject	Age (years)	Sex	Reported hearing loss etiology	Previous style of hearing aids	Previous fitting	Previous hearing aid strategy	Study worn hearing aids
*Adults*							
1100	78	F	Presbycusis	ITC	Bilateral	Digital	Savia 311
1112	69	M	Noise induced	None	None	n/a	Savia 311
1115	60	M	Noise exposure	BTE	Bilateral	Digital	Savia 311
1109	67	M	Acoustic trauma	BTE	Bilateral	Digital	Savia 411
1114	65	F	Unknown	ITC	Bilateral	Analog	Savia 411
1101	68	M	Noise induced	ITC	Monaural	Digital	Savia 311
1111	81	M	Unknown	ITE	Monaural	Digital	Savia 311
1104	59	M	Noise induced, family history	None	None	n/a	Savia 311
1113	81	M	Noise induced	BTE	Bilateral	Digital	Savia 411
1116	50	F	Unknown	BTE	Bilateral	Digital	Savia 411
1108	77	M	Industrial noise exposure	BTE	Bilateral	Digital	Savia 411
1105	71	M	Noise induced	BTE	Bilateral	Analog	Savia 311
1110	67	F	Unknown	BTE	Bilateral	Digital	Savia 411
*Children*							
2061	11	F	Unknown	None	None	n/a	Savia 311
2068	13	M	Unknown	BTE	Bilateral	Digital	Savia 411
2029	12	M	Family history	BTE	Bilateral	Digital	Savia 311
2032	12	F	Ototoxic drugs	BTE	Bilateral	Digital	Savia 311
2069	9	M	Premature birth	BTE	Bilateral	Digital	Savia 411
2062	8	M	Premature birth, ototoxic drugs	BTE	Bilateral	Digital	Savia 311
2034	10	M	Unknown	BTE	Bilateral	Analog	Savia 311
2063	14	M	Ventilator use at birth (respiratory complications)	BTE	Bilateral	Digital	Savia 411
2066	6	M	Asphyxia at birth	BTE	Bilateral	Digital	Savia 411
2060	17	F	Unknown	BTE	Bilateral	Digital	Savia 311
2065	14	F	Oxygen deprivation at birth	BTE	Bilateral	Digital	Savia 411

For fitting, age-dependent prescriptive targets were matched using simulated real ear measures incorporating individual real ear to coupler differences (RECDs). Note that the DSL v5 algorithm prescribes more gain for children than for adults (Scollie et al, 2005). The Audioscan® Verifit VF-1 was used to measure aided responses for speech at 55, 65, and 75 dB SPL, and for a 90 dB SPL tone burst test signal. Fit to targets in the acclimatization phase within 5 dB up to 4000 Hz were obtained for participants with better-ear, high-frequency pure-tone averages (HFPTA) of up to 77 dB HL for the adults and 87 dB HL for the children. Above this, the target gain values could not be achieved, and the upper bandwidth of fit to targets became lower as hearing levels and/or audiometric slopes increased. Target MPO values achieved target at 2000 Hz even for the participants with the greatest hearing loss. Taken together, these fitting results indicate that the hearing aids, although powerful enough for the participants' losses, were affected by bandwidth limitations typical of hearing aid technology, particularly for the participants with greater hearing losses.

### Device fitting with NFC

Custom software was used to enable the NFC processor while holding gain and amplitude compression parameters constant. Two NFC parameters were programmable: (1) the cut-off frequency, which determined the start of the upper band; and (2) the compression ratio, which determined the amount of frequency compression applied to the upper band. The cut-off frequency and compression ratios were determined on an individual basis as follows. The goal was to provide more audibility of high-frequency speech cues, compared to the CP fitting, while limiting negative effects such as poor sound quality or confusion of /s/ with /∫/. The fitter was instructed to evaluate the audibility of the peaks of average-level conversational speech, and of live voice productions of both /s/ and /∫/, on the same display used to fit the hearing aids without NFC. The fitter then enabled NFC and re-evaluated using the same signals to determine if NFC produced an increase in audibility for high-frequency speech energy. Listening checks and aided spectra were used to judge whether the current NFC setting caused confusion and/or spectral overlap of /s/ and /∫/, as judged by the fitter. Fitter judgments were used so that fitting could proceed even if the wearer was too young to participate in the fitting process via subjective comments. Wearer feedback was elicited if the participant could provide it, sometimes resulting in fine tuning. Tuning was most often aimed at reducing the amount of NFC, in response to reports of perceived slurring or excessive audibility of high frequency sounds. In these cases, fitters aimed to provide enough NFC to provide audibility of new sounds without slurring, at an acceptable level. In this process, the better ear was used to select initial NFC settings for both ears, in order to provide symmetrical frequency lowering to the binaural system. Final fittings were symmetrical in all participants (based on better-ear HFPTA values), with the exception of one participant with a significant asymmetry in audiometric thresholds (see Figures 5 and 6 for NFC settings per individual). The fitting for this participant used asymmetrical NFC parameters in order to provide high-frequency audibility per ear.

### Objective outcome measures

Four objective tests were administered (aided speech sound detection, consonant recognition, plural recognition, and vowel recognition, details below). For these tests, stimuli were digitized at a sampling frequency of 48828 Hz and routed through a computer-controlled psychoacoustic workstation (TDT) to the external inputs of a clinical audiometer. The outputs of the audiometer were routed to power amplifiers (R300 for the front speaker, and two Amcron D-75 amplifiers for speakers 2 through 5). The majority of the outcome measures utilized the front speaker only, with the exception of the plural recognition task (described below). The power amplifiers were routed to loudspeakers arranged at 72 degree spacing, one metre from the participants' test location. Participants were seated in an adjustable chair, facing a loudspeaker and computer display, adjusted in height to the level of the loudspeaker.

Aided detection thresholds for speech sounds were measured in the sound field using an adaptive, computer-controlled version of the Ling six-sound test (Ling, 1989; Tenhaaf & Scollie, 2005). The /∫/ and /s/ sounds were selected from this test. These items were spoken by a female talker and recorded and digitized using a studio grade microphone (AKG) coupled to a pre-amplifier and analog to digital converter (USB Pre) and sound recording software (SpectraPlus). The participants selected ‘heard it’ or ‘didn't hear it’ options on the computer monitor. A phoneme-specific detection threshold was bracketed via computer control of programmable attenuators (TDT PA5). Thresholds were estimated as the average level of the last four reversals to a 50% detection criterion, using a 5-dB step size.

The consonant recognition task was a modified version of The University of Western Ontario Distinctive Features Differences test (UWO-DFD) (Cheesman & Jamieson, 1996). The UWO-DFD test was originally developed with four talkers and 21 nonsense disyllables. For the purpose of this study, the task was modified to include a subset of 10 high-frequency consonants: /t∫, d, f, j, k, s, ∫, t, ð, z/. All items were presented in a fixed, word-medial context (i.e. λCIl). Each of the 10 stimuli were spoken by two different female talkers and repeated three times for a total of 60 stimulus presentations.

A plural recognition task was included to assess participant ability to use the fricatives /s/ and /z/ as bound morphemes. Stimuli for this task were chosen to be similar to those used in previous research to test sensitivity to high-frequency audibility in children who use hearing aids (Stelmachowicz et al, 2002). The task included the singular and plural forms of 15 words: ant, balloon, book, butterfly, crab, crayon, cup, dog, fly, flower, frog, pig, skunk, sock, and shoe. These items were recorded using the same talker and procedures used for the Ling 6 stimuli. The female speaker was instructed to speak in a monotonic fashion to avoid inclusion of unnecessary intonation across test items. Four recordings of each item were made, and the token with the least intonation as perceived by the experimenter was selected. The final tokens were equalized in level and had 250 ms of silence added to the beginning and end using sound editing software (Goldwave). The resulting stimuli were presented in the sound field at zero degrees azimuth. Speech-shaped noise was presented simultaneously to the test items at a +20 dB signal-to-noise ratio (SNR) from a clinical audiometer to four other loudspeakers encircling the subject at 72-degrees spacing. The noise was included to mask a low-level stimulus offset cue that could have served as a surrogate cue for plural identification. Pilot data indicated that the +20 dB SNR was adequate for this purpose. Participants selected either the singular or plural form of the target word from a picture and orthographic display on the computer monitor. Two repetitions of all items were presented in random order, for a total of 60 items per score.

A vowel recognition task was included to evaluate whether frequency compression negatively affected vowel perception. Stimuli were selected from a publicly available database of vowels presented in an hVd context, created by Hillenbrand (2003) at Western Michigan University (http://homepages.wmich.edu/~hillenbr/voweldata.html). For the purpose of this study, vowel stimuli included five items (heed, hid, head, had, and hayed), spoken by both adult female and child female talkers. These stimuli had energy at the second formant that spanned the range from roughly 1800 to 3400 Hz, and third formant energy from 2800 to 3700 Hz (Hillenbrand et al, 1995). We expected these frequency regions to be important for vowel perception and likely included in the high band affected by NFC. Each item was presented twice for a total of 20 items per score. Participants selected from one of five orthographic representations on a computer screen.

Presentation level was varied to accommodate the various hearing levels and speech recognition abilities of participants. The minimum testing level was 50 dB SPL, representing speech at a low vocal effort level (Olsen, 1998). The test level was increased if the participant's score for a given test level was at or below chance performance. Increases up to a test level of 65 dB SPL were required for some participants, particularly for the plural identification and consonant recognition tasks. For this reason, comparison of performance across participants in this study was not completed, because test conditions were not held constant. Rather, the relative performance with and without NFC was evaluated, because the test levels were held constant across the final two stages of the trial.

### Real world preference measure

A diary of hearing aid performance was completed in the treatment withdrawal phase of the study. Participants were asked to make direct comparisons between two memories in the hearing aid (with and without NFC). All other aspects of hearing aid processing (i.e. omnidirectional microphone, gain, amplitude compression) were matched between these two programs. The hearing aids automatically started up in program one; therefore listeners were required to use the program switch to select each program for comparison purposes. Participants indicated which program they preferred overall. Participants were given the option of choosing ‘same’ if they felt there was no difference between the programs being compared.

## Results

### Analysis strategies

Two analysis strategies were used. First, an analysis of group-level results was completed. Second, results for individual participants were analysed using single subject design methods, specifically using a modified two standard deviation band technique (Portney & Watkins, 2000). Contributing factors to individual results were explored using multiple regression analysis.

### Group level analyses

#### Speech sound detection

**Figure 2:**
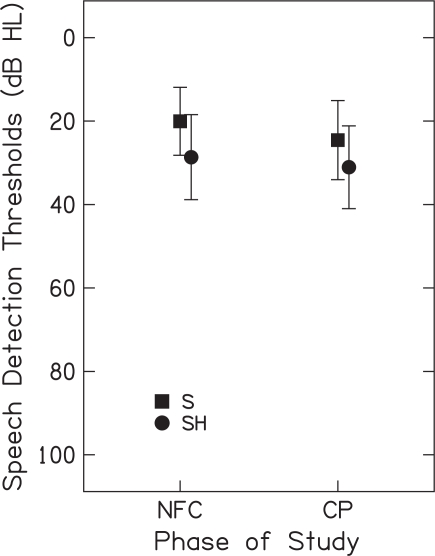
Mean speech sound detection thresholds for adults and children combined, plotted in dB HL for CP (conventional processing) and NFC (nonlinear frequency compression) study phases.

A repeated measures ANOVA was completed with processor type (CP versus NFC) and phoneme (/s/ or /∫/) as within-subject variables, and age group (adults versus children) as a between-subjects variable. Significant simple main effects were found for the processor type as well the phoneme type (F(1,22) = 42.97, p < .001; F(l,22) = 6.84, p = .02). Figure 2 displays mean changes in aided speech sound detection thresholds for each phoneme. These results indicate that the /s/ phoneme had a lower threshold level than did the /∫/ phoneme. Also, aided thresholds were somewhat lower (i.e. better) when the NFC processor was activated.

#### Speech recognition

**Figure 3:**
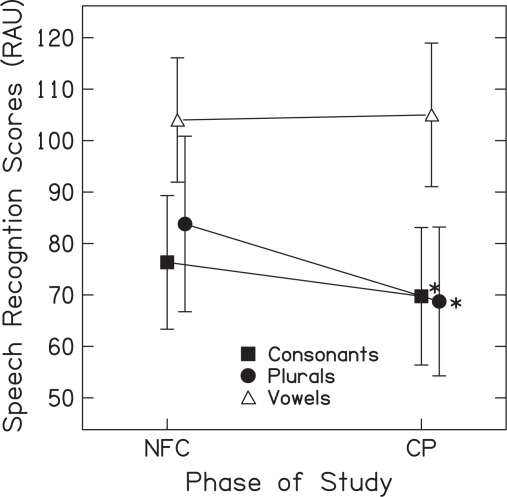
Mean speech recognition scores for adults and children combined, plotted in RAU for CP (conventional processing) and NFC (nonlinear frequency compression) study phases. Statistical significance based on post-hoc analysis at the level of p < .05 is displayed using an asterisk.

A repeated measures ANOVA was completed with processor type (CP versus NFC) and test type (consonant, plural, or vowel recognition) as within-subject variables, and age group (adults versus children) as a between-subjects variable. Mean values were substituted for one participant with missing data on the consonant recognition task. Raw scores for the three speech recognition tasks were converted to rationalized arcsine units (RAU) prior to analysis (Studebaker et al, 1995). Results suggest a significant interaction between test type and processor type (F(2,21) = 8.99, p < .001). Post hoc paired comparisons were conducted with a Bonferroni correction to control familywise error rate to a level of .05. These comparisons indicated that scores were significantly higher with NFC activated, for the consonant and plural recognition tests (t(23) = 3.40, p = .002; t(23) = 5.15, p < .001). Mean speech recognition scores are shown in Figure 3. An asterisk is displayed for pairs of means that differed significantly. These results indicate that, on average, high frequency speech recognition was improved with the use of frequency compression, while vowel perception did not change significantly.

### Single-subject results

Individual scores obtained in the treatment versus withdrawal phases were analysed using confidence limits for performance change. Significant change was deemed to occur when performance in the treatment condition exceeded statistically determined confidence limits. These limits were calculated for levels of significance equivalent to the 90th, 95th, and 99th percentiles.

#### Speech sound detection

**Figure 4:**
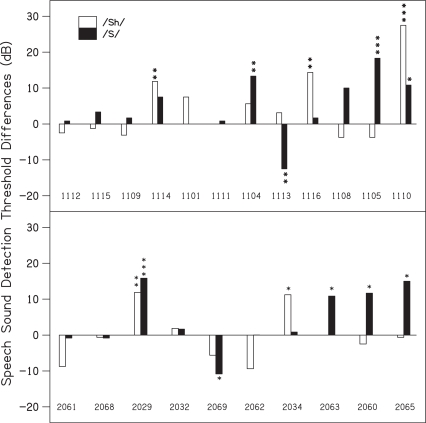
Individual speech sound detection thresholds plotted as difference scores, with a negative score indicating improvement with CP (conventional processing) and a positive score indicating improvement with NFC (nonlinear frequency compression). Results displayed in the top and bottom panes represent the adult and child participants respectively, with participants arranged in order of increasing hearing loss. Statistical significance is shown by asterisk symbols: *p < .10, **p < .05, ***p < .01.

Figure 4 displays individual speech sound detection results plotted as difference scores. Negative scores indicate improvement with CP and positive scores indicate improvement with NFC. For the speech sound detection task, 99%, 95% and 90% confidence limits were computed as ±2.58, ±1.96, and ±1.65 times the standard deviation of test-retest differences across all test stimuli, all participants, in all phases of this trial (SD = 6.04 dB). The 99%, 95% and 90% confidence interval for individual change in speech recognition scores were therefore ±16 dB, ±12 dB and ±10 dB respectively. Two participants (1100, 2066) could not complete the speech sound detection task reliably due to cognitive and/or developmental status; therefore, results for these participants do not appear in Figure 4.

#### Speech recognition

**Figure 5:**
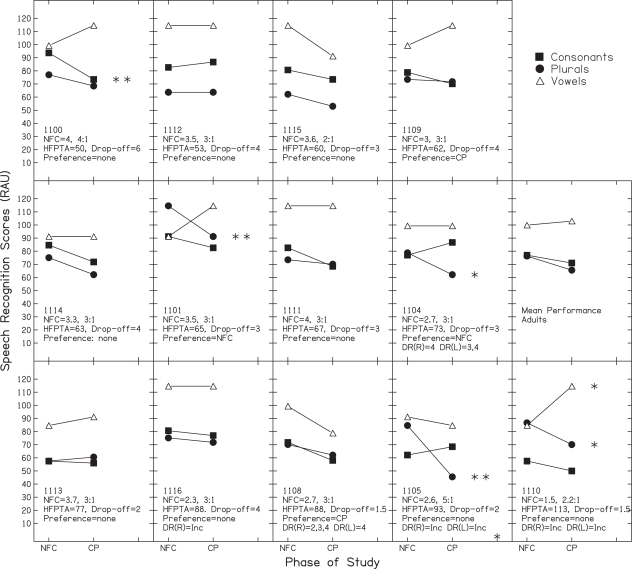
Individual speech recognition results for the adult participants, plotted for treatment (NFC) and treatment withdrawal (CP) study phases. Results are displayed from left to right in order of increasing hearing loss determined by better-ear, high-frequency pure-tone average (HFPTA). Speech recognition scores have been displayed in RAU. Statistical significance is shown by asterisk symbols: *p < .10, **p < .05, ***p < .01. Individual participant figure panes include: subject number, NFC setting (cut-off in kHz, compression ratio), HFPTA (dB HL), hearing loss drop-off (point at which thresholds drop to 70 dB HL, in kHz), self-reported processing preference, and presence of cochlear dead regions (DR) in kHz right (R) and/or (L) side, with ‘Inc’ denoting inconclusive TEN test results.

**Figure 6:**
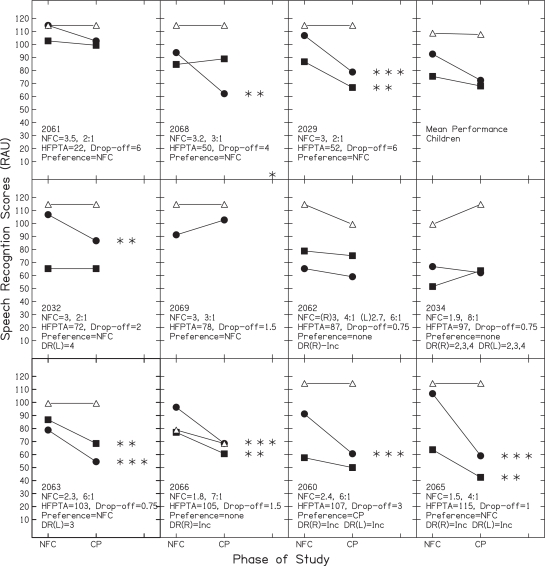
Individual speech recognition results for the child participants, plotted for treatment (NFC) and treatment withdrawal (CP) study phases. Refer to legend on Figure 5. Results are displayed from left to right in order of increasing hearing loss determined by better-ear, high-frequency pure-tone average (HFPTA). Speech recognition scores have been displayed in RAU. Statistical significance is shown by asterisk symbols: *p < .10, **p < .05, ***p < .01. Individual participant figure panes include: subject number, NFC setting (cut-off in kHz, compression ratio), HFPTA (dB HL), hearing loss drop-off (point at which thresholds drop to 70 dB HL, in kHz), self-reported processing preference, and presence of cochlear dead regions (DR) in kHz right (R) and/or (L) side, with ‘Inc’ denoting inconclusive TEN test results.

Figures 5 and 6 display individual speech recognition results plotted for the treatment evaluation (NFC) and treatment withdrawal (CP) phases of the study. In each figure, individual participants have been sorted according to their hearing levels. Scores for the consonant recognition task (60 items), the plural recognition task (60 items), and the vowel recognition task (20 items) are shown for each participant. One child participant (2069) was unable to complete the consonant recognition task due to developmental status. The confidence limits for significant change on each task were calculated based on the binomial theorem, at a score of 50% correct (Raffin & Thornton, 1980). Prior to application of the confidence limits, speech recognition scores were converted to RAU. Conversion to RAU ensured that confidence limits derived at the 50% performance level would be applicable across other performance levels (Studebaker et al, 1995). The 99% confidence limit for individual change in speech recognition scores on the consonant and plural recognition tasks was ±24 RAU, the 95% confidence limit was ±18 RAU, and the 90% confidence limit was ±15 RAU. For the vowel recognition task the 99% confidence limit for individual change was ±41 RAU, the 95% confidence limit was ±31 RAU, and the 90% confidence limit was ±26 RAU. Using these limits, individual changes were judged per task, and significant changes are indicated by the asterisks in the figures.

#### Self-reported preference

Measures of real world preference collected via multi-memory comparison prior to the withdrawal phase are noted per participant in Figures 5 and 6.

### Individual results and contributing factors

Figure 4 through 6 demonstrate variability in benefit received from NFC processing across both adult and child participants included in the study. Trends observed in the data warranted further exploration into the relationships between degree of hearing loss and speech recognition/detection benefit, and age group and speech recognition/detection benefit. Multiple linear regression analyses were completed to investigate the relationships between possible candidacy variables and scores reported across all measures. Three candidacy variables were included: age group, magnitude of high-frequency hearing loss, and audiometric drop-off frequency. Age group was determined by classifying participants as a child (i.e. less than or equal to 18 years) or an adult (i.e. greater than or equal to 19 years). Magnitude of loss was computed using HFPTA. Audiometric drop-off frequency, in kHz, was defined as the frequency at which thresholds met or exceeded 70 dB HL in the better ear. Predictor variables were entered into a stepwise multiple linear regression analysis with backward elimination. The regression was repeated for five measures: consonant recognition, plural recognition, /∫/ detection, /s/ detection, and self-reported preference. Predictors with significant (alpha less than .05) partial correlations were included, and those with nonsignificant partial correlations (alpha greater than .10) were excluded at each step of the analysis. Results for the final step are included in Table 4.

**Table 4:** Final results for multiple linear regression using backward elimination, repeated across measures. Predictor variables are included for plural recognition, /∫/ detection, and /s/ detection tasks, as well as for the self-reported preference measure. Predictor variables include age group, denoted by ‘group’, magnitude of high-frequency hearing loss, denoted by ‘HFPTA’, and audiometric drop-off frequency, denoted by ‘drop-off’. Excluded variables have been removed from the Table. Multiple R squared values are listed for the final equations corresponding to measures with significant findings.

		Unstandardized coefficients		
			
Measure	Predictor variable	b	Standard error	Multiple R squared
Plural recognition	constant	−48.14	23.30	
	group	9.56	5.35	
	HFPTA	0.45	0.19	
	drop-off	5.16	2.75	0.31
/∫/ detection	constant	28.57	12.89	
	HFPTA	0.28	0.11	
	drop-off	−3.38	1.63	0.22
/s/ detection	constant	31.30	10.87	
	HFPTA	−0.33	0.10	
	drop-off	−3.78	1.38	0.36
Preference	constant	1.45	0.39	
	group	0.55	0.26	0.17

For the consonant recognition task, all three predictors were entered into a backward regression analysis and age group as a predictor variable was removed on step one [partial = .11, t(23) = .51, p = .62]; HFPTA as a predictor variable was removed on step two [partial = .35, t(23) = 1.70, p = .10]; audiometric drop-off as a predictor variable was removed on step three [partial = .13, t(23) = .60, p = .55]. None of the candidacy variables predicted benefit on the consonant recognition task. For the plural recognition task, all three predictor variables were entered and all were included in the final regression equation; benefit was significantly predicted by age group, magnitude of high-frequency hearing loss, and audiometric drop-off frequency. For the /∫/ detection task, all three predictors were entered and age group was removed on step one [partial = .30, t(23) = 1.41, p = .18]. Benefit on the /∫/ detection was significantly predicted by magnitude of high-frequency hearing loss and audiometric drop-off frequency. For the /s/ detection task, all three predictor variables were entered and age group was removed on step one [partial = .01, t(23) = .05, p > .96]. Benefit for the /s/ detection task was significantly predicted by magnitude of high-frequency hearing loss and audiometric drop-off frequency. For the self-reported preference measure, all three predictor variables were entered in the backward regression analysis; HFPTA as a predictor variable was removed in step one [partial = −.01, t(23) = −.06, p = .95]; audiometric drop-off was removed in step two [partial = .10, t(23) = .48, p = .64]. Processing preference, as reported on the self-reported preference measure, was significantly predicted by age group in the multiple regression analysis.

In summary, participants with certain hearing losses derived greater NFC benefit on plural recognition and detection tasks. Specifically, participants with a greater amount of high-frequency hearing loss (based on HFPTA) that occurs at higher frequencies (based on the drop-off frequency) derived greater NFC benefit. In addition, there is evidence of a significant age effect for the plural recognition task and the self-reported preference measure. This implies that children deriving greater plural recognition benefit from NFC also had stronger preference for NFC, when compared to the adults.

## Discussion

The main purpose of this study was to examine if prototype multichannel nonlinear frequency compression (NFC) hearing-aid processing provided benefit relative to the same hearing aid fitting without NFC. The NFC processor used in this study was a multichannel (i.e. two-band) strategy that provided frequency lowering (via frequency compression) to the high-frequencies while leaving the low band unaltered in the frequency domain. This was evaluated across a range of participants with varying audiometric characteristics and ages. Results suggest that the NFC processor was, on average, effective at improving high-frequency audibility, resulting in improvements in high-frequency speech sound detection and recognition. No significant changes were observed for low frequency speech sounds (i.e. vowels), on average. Benefit observed from NFC can be attributed to the increased audibility of additional high-frequency energy, albeit presented in a lower frequency range, compared to the conventional hearing aid fittings used in this study. These results generally agree with those reported by Simpson et al (2005) who measured an overall improvement in recognition of high-frequency consonants when a similar NFC processor was used. The results also generally agree with those reported by Miller-Hansen et al (2003), who reported benefits for speech sound detection and recognition with a transposing device.

Analyses performed at the individual level provide evidence that NFC benefit varies across individuals. The results also indicate that the degree of high-frequency hearing loss may predict NFC benefit. This observation agrees with the group findings reported by Miller-Hansen et al (2003), who found greater benefit with a transposition aid for children who had greater hearing losses. In some cases, the individual results presented in this study disagree with the results reported by Simpson et al (2006). Specifically, individuals in this study with high-frequency, steeply sloping losses were more likely to benefit with NFC while those in the Simpson study did not. Participants with steeply sloping losses tested by Simpson et al did not show significant benefit overall, despite having similar losses to the participants included in this study. This may be unexpected, given that the NFC processor in this study was based on the processor proposed by Simpson et al (2005, 2006). However, different fitting methods and hearing instruments were used in the two studies. The present study used a later-generation digital signal processor that offered greater processing power. This change allowed such improvements as more channels of amplitude compression, better control of oscillatory feedback, and provision of a separate device and signal processor per ear, overcoming several technology limitations specifically discussed by Simpson et al (2006).

The individual results also provide evidence that the age of the participants included in this study influenced the degree of NFC benefit. Although mean high-frequency thresholds for adults and children were not significantly different, the mean thresholds were 7 to 10 dB higher for the children in the mid/high frequency range. This difference may have been a factor in outcomes reported in this study. Furthermore, adult and child participants were fitted using different levels of audibility; adults received the DSL v5 adult prescription, which provides 5 to 10 dB less gain than the corresponding paediatric prescription (Scollie et al, 2005). This difference in levels of audibility may have factored into the adult-child differences observed in benefit; children may have received a greater level of audibility for the speech cues that were examined, compared to the adult group. However, Stelmachowicz et al (2004) argue that children require greater audibility than do adults in order to attain equivalent performance on speech recognition tasks. If this is true, the children in this study may have required the audibility of high-frequency cues of speech more so than did the adults and therefore benefitted more from the NFC. For example, the child participant with the mildest level of hearing loss observed in the study performed at ceiling on all speech recognition tasks, but indicated a significant, blinded preference for NFC technology on the grounds that it reduced listening effort in the classroom. This speaks to the heavy listening demands placed on children in educational environments (Crandell & Smaldino, 2000; Hicks & Tharpe, 2002), which may also relate to children's candidacy for NFC. Interpretation of the individual findings may be restricted to the small sample employed in this study: further work to investigate whether age and/or hearing status are predictors of outcome with NFC is needed.

The variability in individual phase durations of this study precludes any speculation as to the time course of acclimatization to NFC processing. However, we would speculate that some time may be required to acclimate to the new audibility provided from NFC, just as occurs for other forms of new audibility (e.g. Gatehouse, 1992; Horwitz & Turner, 1997; Kuk et al, 2003; Munro & Lutman, 2003). It is also possible that our adult and child participants may have varied in their ability to acclimate to the NFC processor. The aging auditory system demonstrates decreases in speech recognition scores and performance on measures of auditory processing and/or cognition, suggesting a decrease in the plasticity of the auditory system with increasing age (Humes & Christopherson, 1991; Humes et al, 1994; [Bibr b11][Bibr b12]). Furthermore, a larger acclimatization effect may be associated with more severe hearing loss (Horwitz & Turner, 1997). The individual results with NFC shown here agree with these suggestions: despite the mean variability in trial durations, the younger participants with more hearing loss seemed to have derived better outcomes. Further research is needed to establish the role of age-related auditory plasticity when measuring benefit change scores, as well as other factors that may be contributing to different rates of auditory acclimatization.

### Relation to clinical practice

These data were collected using prototype hearing instruments, with pre-clinical software that allowed independent manipulation of cut-off frequency and frequency compression ratio per ear and over a wide range of values. A manual fitting approach was used to individualize settings. Since this study was conducted, several similar although not identical clinical hearing instruments have been issued by the manufacturer of these prototype hearing devices (Phonak). These devices use a very similar processing strategy for NFC, with a range of NFC settings that (1) tie the crossover frequency together with the frequency compression ratio; and (2) use a more restricted range of settings than was available in the pre-clinical software. However, the range of settings available clinically is similar to the range actually used in this project (i.e. the pre-clinical software offered a wider range of settings than were actually used). If the clinical hearing devices are fitted to children, the default NFC settings are based on a regression analysis of the pediatric fittings described in this paper; better-ear HFPTA hearing loss values were used as the basis for calculation of NFC settings, which are the same for both ears in the case of bilateral fittings. Clinicians using this technology may choose to employ the more detailed fitting method and/or findings in the present study to better understand one possible method for fitting NFC. Knowledge outcomes presented in this paper may further support fine tuning and troubleshooting of NFC devices.

### Summary

Prototype nonlinear frequency compression (NFC) technology was evaluated for 24 hearing-impaired listeners using various objective and subjective outcome measures. Results can be summarized as follows:

- On average, the NFC processor improved speech sound detection thresholds, as well as consonant and plural recognition scores; vowel perception was not significantly changed. These findings are consistent with the fitting rationale and processor used in the study, which aimed at lowering high-frequency speech energy without affecting low and mid frequency speech energy.
- Individual results indicated that age group and degree and configuration of hearing loss were related to NFC benefit. The following trends were observed: (1) magnitude of high-frequency hearing loss and individual benefit on plural recognition/speech sound detection tasks were related, and (2) audiometric drop-off frequency and individual benefit on plural recognition/speech sound detection tasks were related. Age group was also related to individual benefit on the plural recognition task; children were more likely to benefit compared to adults.
- Individual preference for NFC processing was related to age group and to benefit; children were more likely to have preference for NFC processing than were adults. Also, individual participants were more likely to prefer NFC if they benefited from it.
- Variance in outcome results at the individual level was considerable. Some individuals experienced greater or lesser benefit than the candidacy predictors would lead one to expect. Further research is needed to generalize predictions of candidacy for this technology.

## Abbreviations

ANOVA: Analysis of variance
BTE: Behind-the-ear
CP: Conventional processing
DR: Dead regions
DSL v5.0: Desired sensation level multistage input/output algorithm
HFPTA: High-frequency pure-tone average
HI: Hearing impaired
MPO: Maximum power output
NFC: Nonlinear frequency compression
RAU: Rationalized arcsine units
RECDs: Real ear to coupler differences
SNR: Signal-to-noise ratio
TEN: Threshold equalizing noise
UWO-DFD: University of Western Ontario distinctive features differences test
